# Supplementary material for: A UMLS-based spell checker for natural language processing in vaccine safety
Source: BMC Med Inform Decis Mak. 2007 Feb 12;7:3. doi: 10.1186/1472-6947-7-3 (PMC1805499; doi:10.1186/1472-6947-7-3)
Supplement: Additional file 13 — RAP application directory. Sets up directory for RAP (RDF application for PHP) source code [file 1472-6947-7-3-S13.gz › rap/doc/phpdoc/__filesource/fsource_default__vocabularyRDFS_RES.php.html]

xml version="1.0" encoding="iso-8859-1"?


File Source for RDFS\_RES.php


# Source for file RDFS\_RES.php

Documentation is available at RDFS\_RES.php

```
1. <?PHP

3. // ----------------------------------------------------------------------------------
4. // RDF Vocabulary Description Language 1.0: RDF Schema (RDFS) Vocabulary (ResResource)
5. // ----------------------------------------------------------------------------------
6. // Version                   : 0.9
7. // Authors                   : Daniel Westphal (dawe@gmx.de)
8. //
9. // Description               : Wrapper, defining resources for all terms of the
10. //                               RDF Schema (RDFS).
11. //                               For details about RDF see: http://www.w3.org/TR/rdf-schema/.
12. //                                Using the wrapper allows you to define all aspects of
13. //                             the vocabulary in one spot, simplifing implementation and
14. //                             maintainence.
15. //
16. // ----------------------------------------------------------------------------------
17. // History:
18. // 02-21-2003                 : Initial version
19. // ----------------------------------------------------------------------------------

21. // RDFS concepts


25. class RDFS_RES{

27. function RESOURCE()
28. {
29. return  new ResResource(RDF_SCHEMA_URI . 'Resource');

31. }

33. function LITERAL()
34. {
35. return  new ResResource(RDF_SCHEMA_URI . 'Literal');

37. }

39. function RDFS_CLASS()
40. {
41. return  new ResResource(RDF_SCHEMA_URI . 'Class');

43. }

45. function DATATYPE()
46. {
47. return  new ResResource(RDF_SCHEMA_URI . 'Datatype');

49. }

51. function CONTAINER()
52. {
53. return  new ResResource(RDF_SCHEMA_URI . 'Container');

55. }

57. function CONTAINER_MEMBERSHIP_PROPERTY()
58. {
59. return  new ResResource(RDF_SCHEMA_URI . 'ContainerMembershipProperty');

61. }

63. function SUB_CLASS_OF()
64. {
65. return  new ResResource(RDF_SCHEMA_URI . 'subClassOf');

67. }

69. function SUB_PROPERTY_OF()
70. {
71. return  new ResResource(RDF_SCHEMA_URI . 'subPropertyOf');

73. }

75. function DOMAIN()
76. {
77. return  new ResResource(RDF_SCHEMA_URI . 'domain');

79. }

81. function RANGE()
82. {
83. return  new ResResource(RDF_SCHEMA_URI . 'range');

85. }

87. function LABEL()
88. {
89. return  new ResResource(RDF_SCHEMA_URI . 'label');

91. }

93. function COMMENT()
94. {
95. return  new ResResource(RDF_SCHEMA_URI . 'comment');

97. }

99. function MEMBER()
100. {
101. return  new ResResource(RDF_SCHEMA_URI . 'member');

103. }

105. function SEEALSO()
106. {
107. return  new ResResource(RDF_SCHEMA_URI . 'seeAlso');

109. }

111. function IS_DEFINED_BY()
112. {
113. return  new ResResource(RDF_SCHEMA_URI . 'isDefinedBy');
114. }
115. }
116. ?>
```

Documentation generated on Fri, 17 Dec 2004 16:17:17 +0100 by phpDocumentor 1.3.0RC3
